# Risk factors for unplanned and crash dialysis starts: a protocol for a systematic review and meta-analysis

**DOI:** 10.1186/s13643-016-0297-2

**Published:** 2016-07-19

**Authors:** Amber O. Molnar, Swapnil Hiremath, Pierre A. Brown, Ayub Akbari

**Affiliations:** Division of Nephrology, Department of Medicine, McMaster University, Hamilton, Ontario Canada; Division of Nephrology, Department of Medicine, University of Ottawa, Ottawa, Ontario Canada; Division of Nephrology, The Ottawa Hospital Riverside Campus, 1967 Riverside Drive, Ottawa, K1H 7W9 Ontario Canada

**Keywords:** Systematic review, Unplanned, Crash, Dialysis, Risk factors

## Abstract

**Background:**

Many patients with kidney failure “crash” onto dialysis or initiate dialysis in an unplanned fashion. There are varying definitions, but essentially, a patient is labeled as having a crash dialysis start if he or she has little to no care by a nephrologist prior to starting dialysis. A patient is labeled as having an unplanned dialysis start when he or she starts dialysis with a catheter or during a hospitalization. Given the high prevalence and poor outcomes associated with crash and unplanned dialysis starts, it is important to establish a better understanding of patient risk factors.

**Methods:**

We will conduct a systematic review and meta-analysis with a focus on both crash and unplanned dialysis starts. The first objective will be to determine patient risk factors for crash and unplanned dialysis starts. Secondary objectives will be to determine the most common criteria used to define both crash and unplanned dialysis starts and to determine outcomes associated with crash and unplanned dialysis starts. We will search MEDLINE, EMBASE and Cochrane Library from inception to the present date for all studies that report the characteristics and outcomes of patients who have crash vs. non-crash dialysis starts or unplanned vs. planned dialysis starts. We will also extract from included studies the criteria used to define crash and unplanned dialysis starts. If there are any eligible randomized controlled trials, quality assessment will be performed using the Cochrane Risk of Bias Assessment Tool. Observational studies will be evaluated using the Newcastle-Ottawa Scale. Data will be pooled in meta-analysis if deemed appropriate.

**Discussion:**

The results of this review will inform the design of strategies to help reduce the incidence of crash and unplanned dialysis starts.

**Systematic review registration:**

Prospero CRD42016032916

**Electronic supplementary material:**

The online version of this article (doi:10.1186/s13643-016-0297-2) contains supplementary material, which is available to authorized users.

## Background

Chronic kidney disease (CKD) defined by an estimated glomerular filtration rate (eGFR) <60 ml/min/1.73 m^2^ affects 8.1 % of the American adult population (approximately 16.2 million people) [1, 2]. The estimated prevalence among Canadian adults is lower at 3.1 % (0.73 million adults). However, this figure is much higher (18.6 %) when restricted to Canadian adults ≥65 years [3]. Studies show that the prevalence of CKD in the USA and Canada has increased over the past decade, likely due to a higher prevalence of risk factors for CKD, such as diabetes and hypertension, and an aging population [1–3]. Although showing signs of stabilization, the annual growth of dialysis programs worldwide over the past two decades has ranged between 6 and 12 % [2]. Unfortunately, many patients will “crash” onto dialysis or initiate dialysis in an unplanned fashion.

A patient is labeled as having a crash dialysis start when he or she is referred late to a nephrologist and therefore has minimal or no nephrology care prior to starting dialysis [4]. An unplanned dialysis start is when a patient does not start dialysis using his or her chosen modality, starts dialysis during a hospitalization or, in certain studies, starts dialysis with a central venous catheter (CVC) as opposed to a permanent access (arteriovenous fistula (AVF), arteriovenous graft (AVG), or peritoneal dialysis catheter) [5]. Unfortunately, there is no consensus definition on the exact timing of referral that qualifies a patient as “crashing” onto dialysis. Various studies have used different time cutoffs, ranging from referral to a nephrologist within 90 days to 12 months of dialysis initiation [6–13]. Certain studies have also used a definition that includes the number of nephrologist visits in the year prior to dialysis initiation [6, 7]. The criteria used to define an unplanned dialysis initiation also differ across studies [5].

The prevalence of crash or unplanned dialysis starts varies somewhat in the literature, likely in large part due to inconsistent definitions across studies, but overall, the prevalence is high. Studies have found that 23 to 38 % of patients “crash” onto dialysis [8, 14–16], and 33 to 63 % of patients initiate dialysis in an unplanned fashion [10, 17–22]. Crash or unplanned dialysis starts are both associated with increased patient morbidity and mortality and lower quality-of-life scores [4, 5, 10, 23–26].

Given the high prevalence and poor outcomes associated with crash and unplanned dialysis starts, we plan to conduct a systematic review with the objective of determining patient risk factors. This will hopefully help clinicians better detect at-risk patients and help to design strategies with the aim of minimizing the incidence of crash and unplanned dialysis starts. As well, consensus definitions are needed to help with the conduct of future studies. The systematic review will be conducted in accordance with recommendations from the Preferred Reporting Items for Systematic Review and Meta-Analysis Protocols (PRISMA-P) 2015 statement (see Additional file 1: PRISMA-P + checklist.docx).

## Methods/design

### Objectives

We will conduct a systematic review with a focus on both crash and unplanned dialysis starts. The first objective will be to determine patient risk factors for crash and unplanned dialysis starts (comparator group non-crash or planned dialysis starts, respectively). Secondary objectives will be to determine the most commonly used criteria to define both crash and unplanned dialysis starts and to determine outcomes associated with crash and unplanned dialysis starts. The primary outcome of interest will be mortality. Secondary outcomes of interest will include quality of life, number of hospitalizations, duration of hospitalization, and cost.

### Search strategy

A comprehensive search strategy will be conducted using MEDLINE, EMBASE, and the Cochrane Library with the assistance of a librarian experienced in systematic reviews. A structured search strategy will be based on controlled vocabulary and relevant key terms and will be broad to prioritize sensitivity (see Appendix which details the search strategy utilized). The references of included articles and existing reviews will be scanned for additional studies.

### Study selection

Identified titles and abstracts will be screened by two independent reviewers. If no abstract is available, the full text will be obtained unless the article can be confidently excluded by title alone. If there is any doubt as to whether or not a study can be excluded, a full-text screen will be performed to reduce the likelihood of incorrectly excluding a relevant study. Full-text versions of potentially eligible studies will be obtained and screened independently by each reviewer. Any disagreements will be reconciled by a third party. References will be cataloged using EndNote and study selection will be documented electronically using Excel.

### Inclusion criteria

The literature review will include studies that report the characteristics and outcomes of patients who have crash vs. non-crash dialysis starts or unplanned vs. planned dialysis starts. We will extract from included studies the criteria used to define crash and unplanned dialysis starts. We will include retrospective and prospective studies and interventional studies (non-randomized and randomized). Non-English articles will be included when there is a translator available at our institution. If no translator is available, then the article will be excluded. Databases will be screened from inception to the present date.

### Exclusion criteria

We will exclude studies published only in abstract form, case reports, case series, narrative reviews, editorials, letters, practice guidelines, and animal studies. Studies involving only pediatric patients or combined pediatric and adult patients where the data are not reported separately will be excluded.

### Data extraction

Data will be extracted by two independent reviewers using a standard data abstraction form. A number of variables related to study identification (author, number and location of centers, year of study, year of data collection), and study design (type of study, sample size, included patients) will be included. All criteria used to define crash or unplanned dialysis starts and all reported patient characteristics/risk factors (i.e., age, reason for starting dialysis, blood test results) will be included. For patient characteristics that are continuous variables (i.e., age), the mean (SD) or median (IQR) values for the crash and non-crash or unplanned and planned dialysis groups will be included along with the reported *p* values. For patient characteristics that are categorical variables (i.e., sex) the numbers and/or percentages for the crash and non-crash or unplanned and planned dialysis groups will be included along with the reported *p* values. The association of reported patient characteristics with crash or unplanned dialysis starts will be recorded (adjusted odds ratio, 95 % confidence interval, *p* value). We will also record all potential confounders adjusted for in the analysis (i.e., age, sex, comorbidities).

Data on the following outcomes and their association with crash/non-crash or unplanned/planned dialysis starts will be recorded: mortality, quality of life, number of hospitalizations, duration of hospitalization, and cost. For categorical outcomes, we will record the number and percentage in each group. For continuous outcomes, we will record the mean (SD) or median (IQR) in each group. The association of each outcome with unplanned or crash dialysis starts will be recorded as reported in the study (i.e., odds ratio, hazard ratio, relative risk; 95 % confidence interval and p value). We will also record all potential confounders adjusted for in the analysis. If key data elements are missing in the manuscript of an included study, we will contact the corresponding author of the manuscript for the relevant data.

### Quality assessment

If there are any eligible randomized controlled trials, quality assessment will be performed using the Cochrane Risk of Bias Assessment Tool [27]. Observational studies will be evaluated using the Newcastle-Ottawa Scale. Bias will be assessed at both the outcome and study level.

### Data synthesis and analysis

For all included studies, a detailed description of the results will be provided in table and text form. If enough studies report a particular risk factor for crash or unplanned dialysis starts or outcome associated with crash or unplanned dialysis starts, the data will be pooled in meta-analysis where possible. Our systematic review is meant to be exploratory, so we do not know in advance all of the risk factors, characteristics or outcomes that will be reported. We also do not know if the risk factors, characteristics, or outcomes will be consistently reported across studies. In the event that pooling of data is not feasible due to inadequate information or excessive heterogeneity, we will descriptively report the results for each risk factor and outcome. Heterogeneity will be assessed clinically (i.e., definitions used between studies for crash or unplanned dialysis starts, timing of reporting of outcomes) and statistically using the *I*^2^ statistic [28]. *I*^2^ values of 25, 50, and 75 % correspond to low, medium, and high levels of heterogeneity, respectively. If meta-analyses are deemed suitable, they will be conducted using RevMan Software (pooled odds ratios or mean differences will be reported). Sensitivity analyses will be conducted based on the timing of nephrology referral used to define crash dialysis starts and the criteria used to define unplanned dialysis starts. Data permitting, we will analyze retrospective and prospective cohort studies separately. Also, data permitting, we will perform meta-regression treating study level reported temporary vascular access data as a covariate. Temporary access has been found to be more prevalent among crash dialysis starts and is associated with increased mortality [4, 29].

## Discussion

In this systematic review, we will assess the risk factors for crash and unplanned dialysis starts. We will also determine the association of clinically important outcomes with crash and unplanned dialysis starts and the most commonly used criteria to define crash and unplanned dialysis starts. The prevalence of crash and unplanned dialysis starts is high [5, 8, 10, 14, 16–22], and both are associated with poor patient outcomes. Crash or unplanned dialysis starts are associated with heightened mortality and carry a high cost burden [4, 5]. Therefore, further study of this issue is needed to ultimately improve the care and outcomes of patients with end stage kidney disease.

Two prior systematic reviews and meta-analyses examined outcomes associated with crash starts and showed an increased risk of mortality associated with crash dialysis starts. These published reviews require updating as the most recent one included studies up to 2012. Neither review had a primary objective of determining risk factors for crash dialysis starts [4, 30]. A review paper including studies up to March 2007 summarized the risk factors for late referral or crash dialysis starts [31]. This review requires updating. As well, the authors did not perform a meta-analysis due to heterogeneity of the data. We may encounter similar heterogeneity in our review; however, the inclusion of more recent studies may also yield results that are amenable to pooling. A systematic review on unplanned dialysis starts published in 2009 commented on outcomes associated with unplanned dialysis starts and their economic impact. This review also requires updating and was likely not comprehensive as it only included eight European studies [5].

Our review will utilize a search strategy that minimizes selection bias of studies as best as possible. Given that there are various criteria and terms used to define and label crash and unplanned dialysis starts, we will need to use very broad terms in our search strategy to ensure that all applicable articles are captured. We anticipate that our search will yield a very large number of titles for screening. As well, the nature of the review is quite broad with many data elements to be abstracted. This will complicate the data abstraction process and analysis but is necessary given the exploratory nature of the review. We predict that heterogeneity will limit our ability to pool many of the data elements. We will likely encounter not only heterogeneity in the reporting of outcomes (timing, definitions) but also heterogeneity between studies in terms of how crash and unplanned dialysis starts are defined. The most appropriate definitions for crash and unplanned dialysis starts are currently a matter of debate, which is one of the reasons for this review [4, 5, 32].

Lastly, confounding needs to be considered when interpreting any conclusions drawn from this review. It would be unethical to randomize patients to crash vs. non-crash or unplanned vs. planned dialysis starts. For this reason, we anticipate that all of the studies included in this review will be observational. For example, we may find that anemia is associated with unplanned dialysis starts. However, an association does not necessarily equate to causation. Anemia may simply be associated with other factors that are the true cause for unplanned dialysis starts, such as patient non-adherence. The same concerns will apply to any conclusions with respect to outcomes associated with crash or unplanned dialysis starts. To minimize concerns with respect to confounding, we will abstract unadjusted and adjusted results for all studies as well as the variables that were adjusted for. We will also consider the biological plausibility of causation for any observed associations.

In summary, our systematic review and meta-analysis will provide a better understanding of the risk factors for and outcomes associated with unplanned and crash dialysis starts. The results may help to inform strategies and interventions that would reduce the incidence of crash and unplanned dialysis starts. This review will also determine the most commonly used definitions for crash and unplanned dialysis starts, which will help to guide future studies in this area.

## Abbreviations

AVF, arteriovenous fistula; AVG, arteriovenous graft; CKD, chronic kidney disease; CVC, central venous catheter

## Appendix

### Search strategy

Database: Embase Classic + Embase <1947 to 2015 May 29>, Ovid MEDLINE(R) in-process and other non-indexed citations and Ovid MEDLINE(R) <1946 to present>

Search strategy: June 1, 2015

1. Nephrology/or “Renal Replacement Therapy”/or exp Renal Dialysis/or exp Renal Insufficiency, Chronic/(340678)

2. (nephrolog$ or esrd or end stage renal disease or end stage kidney disease or chronic kidney failure or chronic renal failure or renal replacement therapy or dialysis or hemodialysis or haemodialysis).tw. (380283)

3. 1 or 2 (485466)

4. ((scheduled or emergency or hospital or programmed or nonprogrammed or urgent or nonurgent or acute or suboptimal or optimal or planned or accelerat$) adj5 (start or initiation)).tw. (8352)

5. (unplanned or unscheduled or crash or “not planned”).tw. (36125)

6. ((late or delay$ or defer$ or rate$) adj3 (consul$ or referr$)).tw. (13742)

7. “time to treatment”/(4581)

8. or/4-7 (62467)

9. 3 and 8 (1924)

10. (Nephrology/or exp Renal Insufficiency, Chronic/or “Renal Replacement Therapy”/or exp Renal Dialysis/) and “Referral and Consultation”/(1831)

11. 9 or 10 (3368)

12. 11 use prmz (1291) Medline

13. Nephrology/or “Renal Replacement Therapy”/or exp Renal Dialysis/or exp Renal Insufficiency, Chronic/(340678)

14. (nephrolog$ or esrd or end stage renal disease or end stage kidney disease or chronic kidney failure or chronic renal failure or renal replacement therapy or dialysis or hemodialysis or haemodialysis).tw. (380283)

15. 13 or 14 (485466)

16. ((scheduled or emergency or hospital or programmed or nonprogrammed or urgent or nonurgent or acute or suboptimal or optimal or planned or accelerat$) adj5 (start or initiation)).tw. (8352)

17. (unplanned or unscheduled or crash or “not planned”).tw. (36125)

18. ((late or delay$ or defer$ or rate$) adj3 (consul$ or referr$)).tw. (13742)

19. “time to treatment”/(4581)

20. or/16-19 (62467)

21. 15 and 20 (1924)

22. (Nephrology/or exp Renal Insufficiency, Chronic/or “Renal Replacement Therapy”/or exp Renal Dialysis/) and *“Referral and Consultation”/(513)

23. (Nephrology/or exp Renal Insufficiency, Chronic/or “Renal Replacement Therapy”/or exp Renal Dialysis/) and “Referral and Consultation”/and “time factors”/(255)

24. (Nephrology/or exp Renal Insufficiency, Chronic/or “Renal Replacement Therapy”/or exp Renal Dialysis/) and *time factors/(23)

25. 22 or 23 or 24 (639)

26. or/21-25 (2324)

27. 26 use emczd (1348) Embase

28. 12 or 27 (2639)

29. remove duplicates from 28 (1905)

30. 29 use prmz (1269) Medline (duplicates removed)

31. 29 use emczd (636) Embase (duplicates removed)

## Additional file

Additional file 1: PRISMA-P 2015 checklist. (DOCX 37 kb)
